# Suitable Mouse Model to Study Dynamics of West Nile Virus Infection in *Culex quinquefasciatus* Mosquitoes

**DOI:** 10.3390/tropicalmed9090201

**Published:** 2024-09-02

**Authors:** Lívia Baldon, Silvana de Mendonça, Ellen Santos, Bruno Marçal, Amanda Cupertino de Freitas, Fernanda Rezende, Rafaela Moreira, Viviane Sousa, Sara Comini, Mariana Lima, Flávia Ferreira, João Paulo de Almeida, Emanuele Silva, Siad Amadou, Marcele Rocha, Thiago Leite, Yaovi Todjro, Camila de Carvalho, Viviane Santos, Marta Giovanetti, Luiz Alcantara, Luciano A. Moreira, Alvaro Ferreira

**Affiliations:** 1 Mosquitos Vetores: Endossimbiontes e Interação Patógeno-Vetor, Instituto René Rachou-Fiocruz, Belo Horizonte 30190-002, Brazil; livia.baldon@aluno.fiocruz.br (L.B.); smendonca@aluno.fiocruz.br (S.d.M.); bmarcal@aluno.fiocruz.br (B.M.); acfreitas@aluno.fiocruz.br (A.C.d.F.); fernanda.rezende@fiocruz.br (F.R.); rafaela.moreira@fiocruz.br (R.M.); viviane.pauline@fiocruz.br (V.S.); sara-grangeiro@live.com (S.C.); alveslima.mariana@gmail.com (M.L.); marcelebio@yahoo.com.br (M.R.); giovanetti.marta@gmail.com (M.G.); luiz.alcantara@fiocruz.br (L.A.); luciano.andrade@fiocruz.br (L.A.M.); 2 Departamento de Bioquímica e Imunologia, Instituto de Ciências Biológicas, Universidade Federal de Minas Gerais, 6627-Pampulha, Belo Horizonte 31270-901, Brazil; ellen.caroline24@gmail.com (E.S.); fvianaferreira@gmail.com (F.F.); emanuelegsilva@gmail.com (E.S.); gbadeguetchin@gmail.com (S.A.); thjfl21@gmail.com (T.L.); todjromathias@gmail.com (Y.T.); 3 Laboratório de Ecologia do Adoecimento & Florestas NUPEB/ICEB, Universidade Federal de Ouro Preto, Ouro Preto 35402-163, Brazil; 4 Plataforma de Microscopia e Microanálises de Imagens, Instituto René Rachou-Fiocruz, Belo Horizonte 30190-002, Brazil; camila.decarvalho@fiocruz.br; 5 Plataforma de PCR em Tempo Real, Instituto René Rachou-Fiocruz, Belo Horizonte 30190-002, Brazil; viviane.santos@fiocruz.br; 6 Department of Sciences and Technologies for Sustainable Development and One Health, University of Campus Bio-Medico, 00128 Rome, Italy

**Keywords:** West Nile Virus (WNV), AG129 mouse, *Culex quinquefasciatus*, vector competence

## Abstract

West Nile Virus (WNV) poses a significant global public health threat as a mosquito-borne pathogen. While laboratory mouse models have historically played a crucial role in understanding virus biology, recent research has focused on utilizing immunocompromised models to study arboviruses like dengue and Zika viruses, particularly their interactions with *Aedes aegypti* mosquitoes. However, there has been a shortage of suitable mouse models for investigating WNV and St. Louis encephalitis virus interactions with their primary vectors, *Culex* spp. mosquitoes. Here, we establish the AG129 mouse (IFN α/β/γ R^−/−^) as an effective vertebrate model for examining mosquito–WNV interactions. Following intraperitoneal injection, AG129 mice exhibited transient viremia lasting several days, peaking on the second or third day post-infection, which is sufficient to infect *Culex quinquefasciatus* mosquitoes during a blood meal. We also observed WNV replication in the midgut and dissemination to other tissues, including the fat body, in infected mosquitoes. Notably, infectious virions were present in the saliva of a viremic AG129 mouse 16 days post-exposure, indicating successful transmission capacity. These findings highlight the utility of AG129 mice for studying vector competence and WNV–mosquito interactions.

## 1. Introduction

West Nile Virus (WNV) is a significant public health concern worldwide, posing serious threats to both human and animal health [1]. First identified in the West Nile district of Uganda in 1937, WNV has since emerged as a major pathogen globally, primarily due to its efficient transmission via mosquito vectors and a wide range of avian and mammalian hosts [2]. Over the past few decades, WNV has demonstrated remarkable adaptability and geographic expansion [3,4]. In the late 1990s, the virus made a notable leap into North America, leading to widespread outbreaks across the United States and Canada [3,4,5]. From there, it continued its spread into South America, further establishing its presence in new ecological niches [5,6]. The extensive distribution and frequent outbreaks of WNV underscore its global public health impact, necessitating robust surveillance, prevention, and control strategies to mitigate its spread and associated morbidity and mortality [1].

WNV belongs to the Flaviviridae family, which includes several notable human pathogens such as dengue virus (DENV), Zika virus (ZIKV), and yellow fever virus (YFV). Structurally, WNV is an enveloped virus with an icosahedral capsid and a lipid bilayer derived from the host cell membrane [7,8] (Figure S1). The virus’s envelope is embedded with two surface glycoproteins, E (envelope) and M (membrane), which are crucial for viral entry into host cells and immune system evasion [7,8,9]. The WNV genome consists of a single-stranded, positive-sense RNA of approximately 11,000 nucleotides in length [9,10]. This RNA genome encodes a single polyprotein that is cleaved by viral and host proteases into three structural proteins (C, prM, and E) and seven nonstructural proteins (NS1, NS2A, NS2B, NS3, NS4A, NS4B, and NS5) [10]. These nonstructural proteins are involved in viral replication, assembly, and modulation of the host immune response [10,11].

WNV exhibits complex transmission cycles that can involve multiple hosts and vectors [2]. The transmission dynamics of WNV are intricately linked to its vector ecology and reservoir hosts [2]. Mosquitoes, particularly species of the *Culex* genus, serve as primary vectors for WNV transmission [2,12]. Among these, *Culex quinquefasciatus*, commonly known as the Southern House Mosquito, plays a significant role due to its widespread distribution and preference for urban habitats where human–mosquito interactions are frequent [13,14]. This vector species thrives in diverse environmental conditions, contributing to the resilience and persistence of WNV transmission cycles in both urban and rural settings [10,14]. Additionally, various avian species, particularly passerine birds, serve as amplifying hosts for WNV, allowing for sustained viral circulation within bird-mosquito transmission cycles [15,16,17]. Importantly, spillover events from these avian reservoirs into mammalian hosts, including humans and horses, can result in severe neurological disease manifestations, highlighting the zoonotic potential and public health implications of WNV transmission [2,9,18].

Infected *Culex* spp. mosquitoes can transmit WNV to humans and other vertebrates during their feeding process [2]. The successful infection of a vertebrate host by WNV is influenced by viral factors, such as genetic variability that affects infectivity and virulence, and host factors, including genetic susceptibility and the physiological state of the host [19,20,21]. Additionally, factors derived from mosquitoes, particularly components found in mosquito saliva delivered to the vertebrate host during blood feeding, can intensify the pathogenesis of these viruses [22,23,24,25]. This underscores the significance of arbovirus research within invertebrate vectors. Furthermore, animal models are indispensable for elucidating human viral infections and pathogenesis. A suitable animal model is essential to enhance our understanding of arbovirus pathogenesis [18,26,27]. To date, various animal models have been employed to investigate arbovirus pathogenesis, yet numerous challenges persist [28,29,30,31,32]. 

Although artificial blood meals are often used as a stand-in for natural feeding, they do not entirely replicate the complexity of a mosquito feeding on a live vertebrate host. Blood meals are the main pathway for mosquitoes to acquire arbovirus infections, and components of the blood or their metabolites may act as host factors that influence mosquito susceptibility to these viruses [33,34,35]. Recent studies indicate that blood components can modulate immune responses, thereby impacting mosquito infection rates [34,35,36,37]. Over recent decades, various immunocompromised knockout mouse strains, such as AG129, have been developed and utilized in arbovirus research due to their lack of both alpha/beta and gamma interferon receptors (IFN α, β, γ R^−/−^) [28,30,31,38].

However, most of these studies have focused on mosquito vectors from the Aedes genus such as Aedes aegypti and Aedes albopictus [38]. In the present study, we investigated the suitability and usefulness of the AG129 mouse model for examining aspects of *Culex quinquefasciatus* vector competence for WNV as well as host factors that influence the extrinsic incubation period and other aspects of mosquito biology that can affect virus–host interactions. We found that intraperitoneal injection of WNV caused systemic infections in AG129 mice, which presented with high viremia. Additionally, we observed that *Culex quinquefasciatus* mosquitoes became infected after feeding on viremic AG129 mice. Furthermore, we demonstrated that with this blood meal approach, WNV was not only able to infect the epithelial cells of the midgut but also overcome the midgut escape barrier, thus disseminating to other tissues in the body cavity. Finally, we observed that WNV was able to reach the salivary glands, and infectious virion particles were secreted in the saliva of *Culex quinquefasciatus* females.

## 2. Materials and Methods

### 2.1. Mice Lineages

For this study, we used AG129 mice (IFN α/β/γ R^−/−^), a double knockout strain that is immunocompromised due to the absence of receptors for type I (IFN α/β) and type II (IFN γ) interferons [38]. These mice were bred and maintained at the Animal Facility of Instituto René Rachou, Fiocruz Minas. All procedures were approved by the Institutional Animal Care and Use Committee, Comissão de Ética no Uso de Animais da Fiocruz (CEUA), and adhered to institutional guidelines (license number LW-26-2).

### 2.2. Mosquito Lineages and Mosquito Rearing

We used *Culex quinquefasciatus* mosquitoes from the Pernambuco (Brazil) laboratory strain, which has been maintained in the laboratory for 148 generations. They were reared under controlled conditions (28 °C, 70–80% humidity, 12/12 h light/dark cycle). Eggs were incubated in plastic trays with two liters of filtered tap water and fish food (Tetramin, Tetra) to facilitate hatching. Larvae were reared at a density of 200 per tray. After emergence, adults were housed in 30 cm × 30 cm × 30 cm BugDorm insect cages and provided with a 10% sucrose solution ad libitum.

### 2.3. Virus Propagation and Titration

In this study, we used the West Nile Virus (WNV) NY99-4132 reference strain (GenBank accession: MH643887), originally isolated from the brain of an American Crow. The virus was propagated in Aedes albopictus C6/36 cells, maintained in Leibovitz’s L-15 medium supplemented with 10% fetal bovine serum (FBS) and 1× Antibiotic-Antimycotic (Gibco, Thermo Fisher Scientific, Waltham, MA, USA). Cells were infected at a multiplicity of infection (MOI) of 0.05 and incubated at 28 °C for five days. The supernatants were collected, clarified by centrifugation, and stored at −80 °C as virus stocks. Mock supernatants were similarly prepared without viral infection. The virus titer was determined by plaque assay in BHK-21 cells. Briefly, BHK-21 cells were seeded in six-well plates, and after virus adsorption for 1 h at 37 °C, the cells were overlaid with 2% carboxymethyl cellulose (CMC) in DMEM containing 2% FBS. Plates were incubated at 37 °C with 5% CO_2_ for five days, fixed with formaldehyde, and stained with a crystal violet solution (70% water, 30% methanol, 0.25% crystal violet) to visualize plaques.

### 2.4. Mice Inoculation with WNV

WNV inoculation of AG129 mice was accomplished by intraperitoneal injection (IP). Three to four-week-old animals were challenged with approximately 8.6 × 10^8^ plaque-forming units (p.f.u.) of WNV per mouse. After inoculation, the mice were observed daily and assessed for signs of morbidity and mortality. For the viremia kinetics studies involving AG129 mice, approximately 0.2 mL blood samples were taken at each time point from individual mice, which were euthanized following each collection. Blood samples were drawn from the tail vein of anesthetized mice. All procedures were carried out in a specific-pathogen-free facility at Instituto René Rachou. The mice were kept in an environment with controlled temperature and humidity, on a 12-h light/dark cycle, with unrestricted access to food and water. Male AG129 mice were used for all experiments.

### 2.5. Mosquito Infection with WNV

Seven to ten-day-old female mosquitoes were placed into cylindrical containers with nylon mesh (0.88 mm hole size) on top and were starved by withholding sugar for 24 h before feeding on mice. All mosquito infections were conducted using AG129 mice. Infected AG129 mice, three days after WNV inoculation, were anesthetized with a ketamine/xylazine mixture (80/8 mg per kg). The anesthetized mice were then positioned on top of the containers covered with netting, allowing the female mosquitoes access. The mice, unshaved and placed prone, had their entire ventral surface and limbs exposed for feeding. The mosquitoes were allowed to feed for 30 min. After feeding, the fully engorged females were selected and transferred to a new container with nylon mesh and provided with a cotton pad soaked in a 10% glucose solution and a plastic cup with soaked paper for egg laying. The female mosquitoes that had fed on the mice were collected individually for RNA extraction eight days after feeding.

### 2.6. Mosquito Salivation and Saliva Infectivity Test

For saliva collection, we used a protocol similar to that of Anderson, Richards, and Smartt (2010) [39]. Briefly, saliva was collected 16 days post-infection (d.p.i.) from mosquitoes (after their legs and wings were removed) by inserting their proboscis into sterile P10 microcapillary tips containing 5 μL of a solution comprising fetal bovine serum and 30% sucrose for 30 min at room temperature. The contents of the tips were then independently expelled into a 0.2 mL tube and stored at −80 °C until injected into other mosquitoes. Abdomens, heads, and thoraxes were independently dissected and homogenized in 200 μL of Trizol reagent (Invitrogen, Carlsbad, CA, USA) and stored at −80 °C until RNA extraction. Saliva from each mosquito was nano-injected into five female *Culex quinquefasciatus* mosquitoes (Pernambuco lineage). Each individual female mosquito was injected with 100 nL of saliva using a Nanojet III (Drummond Scientific, Broomall, PA, USA). Injection was performed in the paratergite region of the mosquito thorax. After injection, recovered females were selected and harvested 5 dpi individually for RNA extraction and WNV detection by RT-qPCR.

### 2.7. RNA Extraction and RT-qPCR

RNA was extracted from whole mosquito specimens or from dissected abdomen and thorax + head parts using the Trizol reagent (Invitrogen, Carlsbad, CA, USA). The extraction process followed the manufacturer’s protocol with minor adjustments. In brief, the samples were placed into 1.5 mL Eppendorf tubes, to which 200 µL of Trizol and two glass beads were added. The tubes were then subjected to vigorous grinding in a bead beater for 90 s. Following this, the samples were incubated at room temperature for 10 min before adding 40 µL of chloroform and mixing the solution vigorously for 30 s. After an additional 10-min incubation at room temperature, the samples were centrifuged at 12,000× *g* for 15 min at 4 °C. The aqueous phase was mixed with an equal volume of chilled isopropanol by inversion for 2 min and stored overnight at −20 °C. The next day, samples were centrifuged at 12,000× *g* for 5 min at 4 °C, and the resulting pellet was washed with 75% ethanol before being air-dried for approximately 10 min. The RNA pellet was then dissolved in 20 µL of RNase-free water and stored at −80 °C.

For cDNA synthesis, total RNA was reverse transcribed using M-MLV reverse transcriptase (Promega, Madison, WI, USA) with random primers for initiation. Negative controls were prepared by omitting the reverse transcriptase. Real-time PCR was carried out using the QuantStudio 12K Real-Time PCR System (Applied Biosystems, Foster City, CA, USA) with the SYBR Green PCR Master Mix (Applied Biosystems—Life Technologies, Foster City, CA, USA). Each reaction had a final volume of 10 µL. The PCR cycling conditions were as follows: Initial hold at 95 °C for 20 s; PCR amplification for 40 cycles at 95 °C for 15 s followed by 60 °C for 60 s; and a melting curve analysis from 60 °C to 95 °C, with a final 15-s hold at each temperature point. All reactions were performed in triplicate.

Gene expression was quantified using the 2^−ΔCt^ method. The Ct (cycle threshold) values for the target gene (WNV gene) were normalized to the Ct values of the internal reference gene (housekeeping gene 18S) within the same sample. The viral RNA levels were reported relative to the endogenous control gene 18S for *Culex quinquefasciatus* and RPL32 for AG129 mice [40,41,42,43].

For *Culex quinquefasciatus*, the 18S primers were as follows: Forward: 5′-CGC GGT AAT TCC AGC TCC ACT A-3′ and Reverse: 5′-GCA TCA AGC GCC ACC ATA TAG G-3′. For WNV, the primers were as follows: Forward: 5′-AAC CKC CAG AAG GAG TSA AR-3′ and Reverse: 5′-AGC YTC RAA CTC CAG RAA GC-3′ or Forward: 5′-CAG ACC ACG CTA CGG CG-3′ and Reverse: 5′-CTA GGG CCG CGT CGT GGG-3′. For mice, the RPL32 primers were as follows: Forward: 5′-GCT GCC ATC TGT TTT ACG G-3′ and Reverse: 5′-TGA CTG GTG CCT GAT GAA CT-3′. Our previous studies have estimated the detection limit for the SYBR Green RT-qPCR assay for WNV to be 0.1 PFU/mL, which is equivalent to −6 Log10.

### 2.8. WNV Immunostaining and Microscopy

Female *Culex quinquefasciatus* mosquitoes were dissected to expose internal tissues. The tissues were fixed in 4% paraformaldehyde in phosphate-buffered saline (PBS) for 10 min, followed by washing in PBS. The samples were then incubated with 1% Triton-X-100 and 5% fetal bovine serum (FBS) in PBS (PTX-FBS) for 30 min. Next, the samples were incubated overnight at 4 °C with the primary antibody 4G2 [44], diluted 1:50. After primary antibody incubation, the samples were washed three times for 15 min each with PTX-FBS. Subsequently, the samples were incubated with secondary antibodies conjugated with Alexa Fluor 488 (Molecular Probes, Thermo Fisher Scientific, Eugene, OR, USA) in PTX-FBS for 2 h. Following secondary antibody incubation, the samples were washed again in PTX-FBS and then incubated with Alexa Fluor 568 Phalloidin and Hoechst 33342 (Molecular Probes) for 15 min. The samples underwent three additional washes, 15 min each, in PTX-FBS before being dissected and mounted in Vectashield Mounting Medium for microscopy. Confocal images were captured using Laser Confocal Nikon C2 + (Nikon Healthcare, Tokyo, Japan) microscope and processed using ImajeJ software (ImajeJ v.1.54f).

## 3. Results

### 3.1. AG129 Mice Exhibit High Susceptibility to WNV Infection

**Figure 1:**
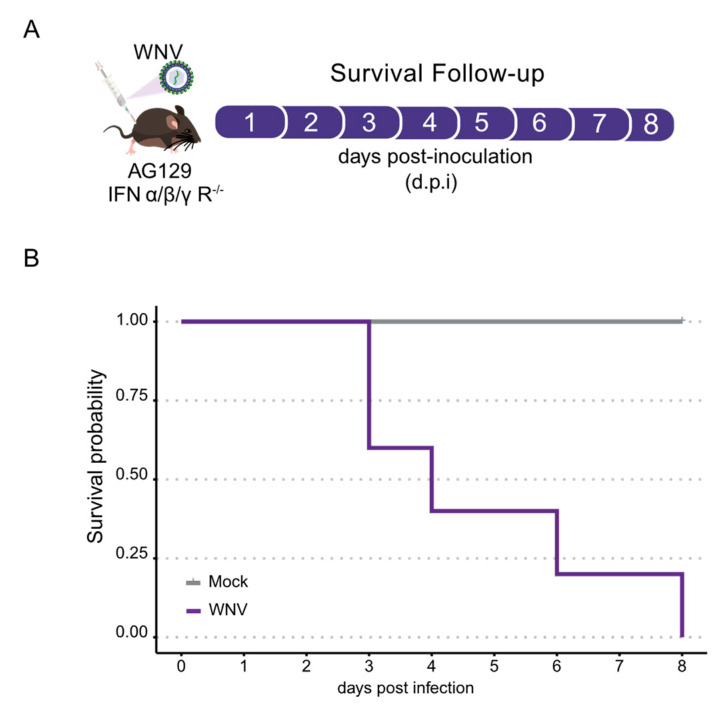
Mortality of immunocompromised AG129 (IFNα/β/γR^−/−^) mice following WNV inoculation. (**A**) Experimental Design: Three or eight-week-old AG129 mice were intraperitoneally inoculated with WNV, with injections performed in the lower right quadrant. (**B**) Kaplan–Meier survival plot depicting the survival probability of three-week-old AG129 mice inoculated with 8.6 × 10^8^ plaque-forming units (p.f.u.) of WNV over eight days.

To assess the susceptibility of AG129 mice to WNV infection, adult mice were intraperitoneally challenged with 8.6 × 10^8^ plaque-forming units (p.f.u.) of WNV per mouse (Figure 1A). We found that AG129 mice began to succumb to the infection three days post-inoculation, resulting in 100% mortality within eight days (Figure 1B).

**Figure 2:**
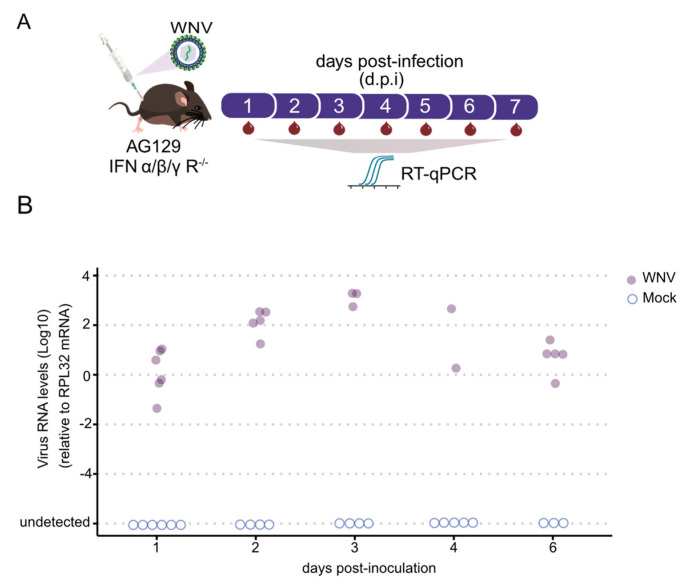
Arbovirus viremia in AG129 mice. (**A**) Experimental setup: Three-week-old AG129 mice were given intraperitoneal injections of WNV in the lower right quadrant. Blood samples were taken every 24 h for eight days to quantify viral RNA using RT-qPCR. Each data point represents a blood sample from an individual mouse, with samples from at least two different mice collected at each time point. Mice were observed daily for eight days and euthanized after one blood collection. (**B**) Viral RNA levels in the blood of AG129 mice inoculated with 8.6 × 10^8^ plaque-forming units (p.f.u.) of WNV.

The subsequent objective was to determine if AG129 mice develop viremia upon WNV infection, thereby establishing them as a vertebrate animal model for this mosquito-borne arbovirus. As illustrated in Figure 2A, we tracked the kinetics of blood viremia by daily estimation of viral load using RT-qPCR. As early as one day post-inoculation, viral RNA was detectable. By two days post-infection (d.p.i.), a significant increase in WNV RNA levels was observed, with all animals displaying detectable viral RNA in their blood (Figure 2B). RNA levels of WNV peaked at day 3, indicating peak viral presence in the blood. Subsequently, RNA levels began to decrease, continuing to decline until day 6 (Figure 2B). This observation led us to expose the mice to *Culex quinquefasciatus* mosquitoes for blood feeding on day 3. Taken together, these results demonstrate that AG129 juvenile mice are highly susceptible to WNV infection.

### 3.2. AG129 Mice Are Capable of Infecting Culex quinquefasciatus Mosquitoes with WNV through Blood Meals

**Figure 3:**
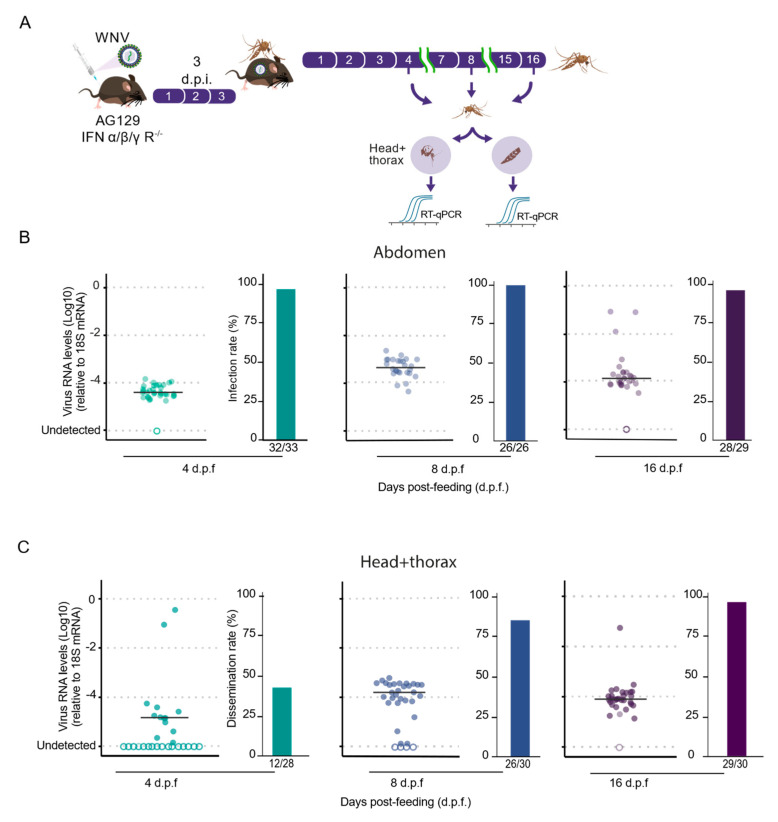
Viremic AG129 Mice as a competent vertebrate animal model for WNV infection in *Culex quinquefasciatus*. (**A**) Experimental design scheme. (**B**) The WNV RNA levels of abdomen of each mosquito tested are shown 4, 8, and 16 days post-feeding (d.p.f.) are shown. Detection of WNV in the abdomen was utilized as an indicator of infection. (**C**) WNV RNA levels in the head + thorax of each tested mosquito at 4, 8, and 16 d.p.f. are shown. Detection of WNV in the head + thorax was utilized as an indicator of dissemination efficiency. The number of mosquitoes with detected WNV RNA out of the total tested is indicated below the bar charts. WNV load quantification was performed using the 2^−ΔCt^ (delta Ct) method. The same individuals from the cohort were used to obtain results in both the abdomen and head + thorax. Empty circles indicate samples where viral RNA was not detected.

The ability of AG129 mice to rapidly develop high levels of WNV viremia shortly after inoculation highlights their suitability as an animal model for investigating vector competence and interactions between *Culex quinquefasciatus* and WNV. To test this hypothesis, female Culex mosquitoes were allowed to feed on viremic AG129 mice (Figure 3A), which were three weeks old at the time of virus inoculation. Using this setup, we evaluated the infection rates of *Culex quinquefasciatus* mosquitoes for the WNV NY99-4132 strain. Seven-day-old female mosquitoes were exposed to the same WNV-infected AG129 mice for blood-feeding for 30 min.

Mosquitoes were collected at four, eight, and sixteen days post-feeding (d.p.f.) to analyze the presence of WNV in the abdomen and head + thorax, assessing infection rates and dissemination efficiency, respectively. The infection rates were determined by calculating the proportion of blood-fed mosquitoes that tested positive for the virus in the midgut relative to the total number of blood-fed mosquitoes. We observed consistently high rates across all three time points, ranging from 96% at 4 d.p.f. to 100% at 8 d.p.f. and 96% at 16 d.p.f. (Figure 3B,C). Remarkably, as shown in Figure 3B, a high infection rate remained at 16 d.p.f., indicating that the mosquitoes were unable to clear the WNV infection from the abdomen region. Next, to analyze the dissemination efficiency of WNV in *Culex quinquefasciatus* mosquitoes after feeding on infectious AG129 mice, we assessed the percentage of mosquitoes in which the virus was detected in their head + thorax, indicating successful escape from the midgut barrier and dissemination into the mosquito body cavity. We observed that WNV was already present in the head + thorax as early as 4 d.p.f. in 43% of the mosquitoes tested (Figure 3A,C). However, the percentage of mosquitoes with disseminated virus increased at both the 8- and 16-day d.p.f. time points, reaching 87% and 96%, respectively (Figure 3C).

**Figure 4:**
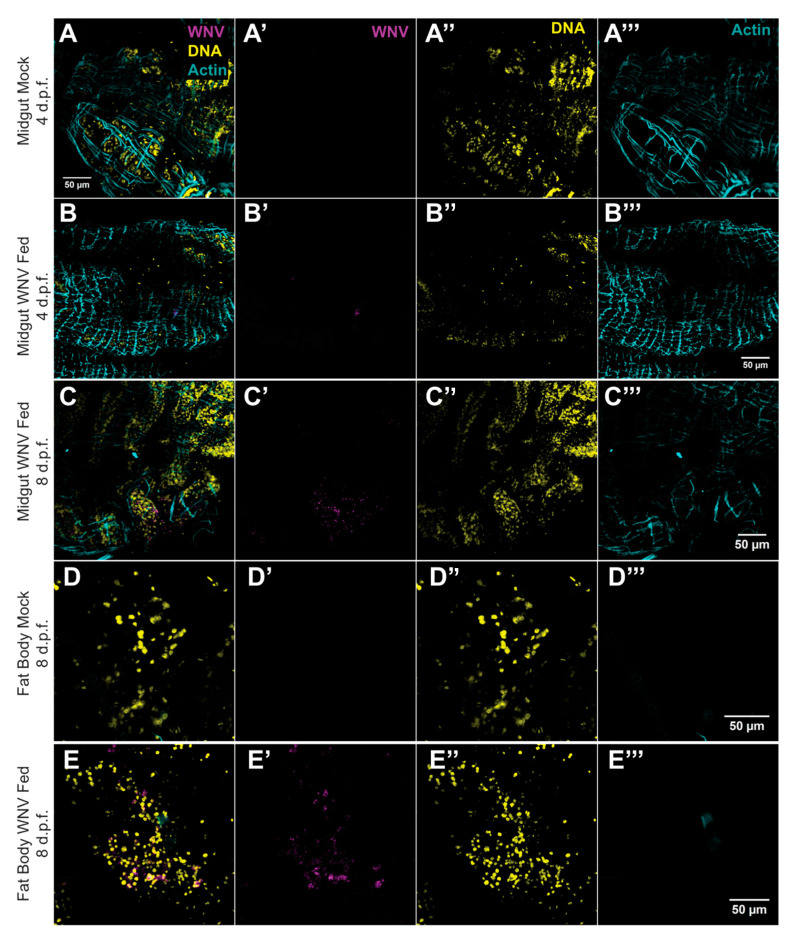
WNV tissue tropism following oral infection in *Culex quinquefasciatus*. (**A**) Midgut of a female mosquito that was fed only on blood. WNV is detected in midgut epithelial enterocytes of female mosquitoes at 4 (**B**) and 8 (**C**) days after feeding on blood from an infectious AG129 mouse. (**D**) Fat body from a female mosquito fed solely on blood. (**E**) WNV was identified in the fat body of female mosquitoes 8 days after feeding on blood from an infectious AG129 mouse. Panels (**A**–**E**) show merged images from triple-stained immunofluorescence assays of adult female tissues. The tissues were stained with an antibody against WNV (magenta), actin marked with phalloidin (cyan), and DNA marked with Hoechst (yellow). (**A′**–**E′**) show WNV immunostaining. (**A″**–**E″**) show DNA staining. (**A‴**–**E‴**) show actin staining.

To better explore the dissemination of WNV within the body cavity and identify infected tissues, we conducted immunostaining to visualize the distribution of viral antigens, which served as an indicator of viral infection location. This approach provided insights into the spread and localization of WNV beyond the midgut in *Culex quinquefasciatus* mosquitoes. Immunofluorescence analysis of WNV-infected female mosquitoes at 4 d.p.f. showed the virus present in the midgut, albeit at very low levels (Figure 4B). By 4 d.p.f., infection was exclusively detected in the midgut, confined to specific regions of the midgut epithelium (Figure 4B). However, by 8 d.p.f., WNV was extensively detected in the midgut, indicating replication within the midgut epithelium before spreading into the body cavity (Figure 4C). Despite WNV’s presence in the midgut epithelium, no virus was detected in the visceral muscle (Figure 4C). Importantly, analysis at 8 d.p.f. also revealed WNV in several fat body regions of female mosquitoes (Figure 4E). This finding underscores the virus’s ability to escape the midgut and disseminate to other tissues, including the fat body, highlighting potential implications for viral persistence and transmission dynamics within the mosquito vector.

### 3.3. High Transmission Efficiency of WNV by Culex quinquefasciatus Mosquitoes Following Blood Feeding on AG129 Infectious Mice

**Figure 5:**
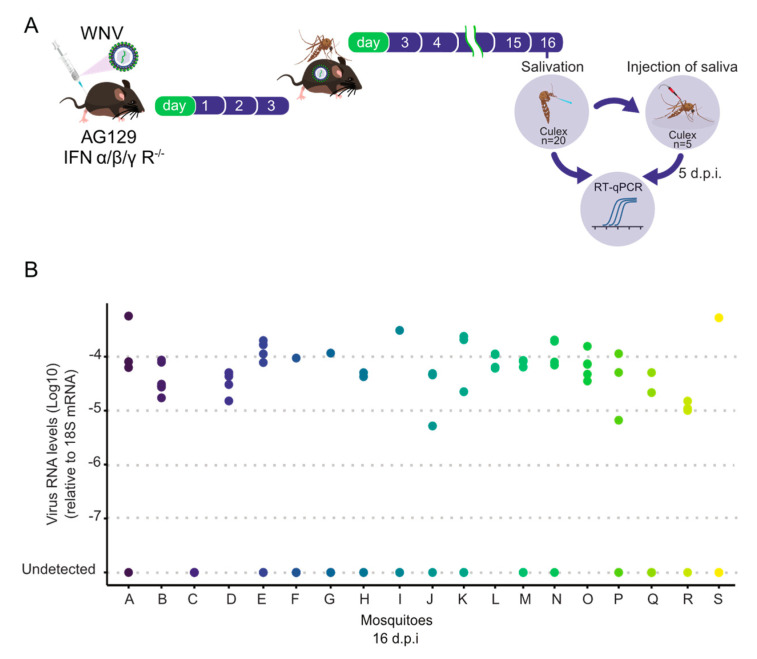
*Culex quinquefasciatus* mosquitoes are capable of transmitting WNV 16 days post-feeding on an AG129 viremic mouse. (**A**) Experimental design scheme illustrating how the infectivity of mosquito saliva was tested. Saliva samples were collected from female mosquitoes (**A**) that had previously taken an infectious blood meal from a WNV-viremic AG129 mouse at 16 d.p.f. The collected saliva from each individual mosquito was then injected into five naïve mosquitoes, which were subsequently tested for WNV infection by RT-qPCR five days post-injection. (**B**) Levels of WNV RNA detected in mosquitoes injected with saliva from 16 d.p.f. females. Mosquitoes that became infected are shown as solid dots, while uninfected mosquitoes are depicted as empty dots. Each letter on the *X*-axis represents a single saliva sample.

Assessing a mosquito’s ability to transmit an arbovirus is crucial both for laboratory vector competence assays and for studies investigating virus–host interactions. To assess the transmission efficiency of WNV by *Culex quinquefasciatus* mosquitoes following blood feeding on AG129 infectious mice, thereby completing the transmission cycle, we analyzed the infectivity of their saliva. For this purpose, we injected undiluted 100 nL of saliva from each mosquito into five naïve *Culex quinquefasciatus* females (mosquitoes that had never previously encountered arboviruses). Afterward, injected mosquitoes were collected five days post-injection, and their whole bodies were subsequently individually examined to analyze the presence of WNV viral RNA by RT-qPCR (Figure 5A). As shown in Figure 5B, we found that 19 out of 20 (95%) saliva samples were able to infect naïve mosquitoes. Among the infectious saliva samples, the number of infected mosquitoes ranged from 1 to 5. Despite the observed variations in the number of mosquitoes infected after saliva injection, it is crucial to note that even if a single mosquito out of five became infected, it indicates the presence of infectious WNV virions in the saliva. Overall, these data demonstrate a high transmission efficiency of WNV by *Culex quinquefasciatus* mosquitoes following blood feeding on WNV-infected AG129 mice.

## 4. Discussion

In this study, we comprehensively evaluated the interactions between WNV, AG129 mice, and *Culex quinquefasciatus* mosquitoes. Our findings underscore the importance of using a live vertebrate host, such as AG129 mice, to fully mimic the natural complexity of blood feeding, which is crucial for understanding the virus’s transmission dynamics. The AG129 mouse model, characterized by its high susceptibility to WNV infection, demonstrated a rapid onset of viremia and high mortality rates. This makes AG129 mice an invaluable model for studying WNV pathogenesis and vector competence. Our results showed that AG129 mice, when intraperitoneally challenged with WNV, developed detectable viremia as early as one day post-inoculation, with peak viral RNA levels observed by day 3. This rapid development of high viremia levels highlights the model’s utility in providing a consistent source of infectious blood meals for mosquitoes.

A key objective of our study was to assess whether the viremia observed in AG129 mice was adequate to infect female mosquitoes with WNV through blood meals. It was essential to evaluate both the viral load and infection rates of WNV. When *Culex quinquefasciatus* mosquitoes were exposed to viremic AG129 mice and the infection status of each female that fed was individually recorded, we found a high infection rate. These results are consistent with previous research on flaviviruses such as DENV and ZIKV, which has shown that viremic AG129 mice can effectively transmit these viruses to mosquitoes via blood meals [38]. Our results with WNV confirm that AG129 mice are suitable for transmitting WNV and consequently infecting *Culex quinquefasciatus* mosquitoes after blood-feeding. Our study demonstrated that female mosquitoes feeding on viremic AG129 mice had high midgut infection rates (96% at 4 d.p.f., 100% at 8 d.p.f., and 96% at 16 d.p.f.), with the virus successfully disseminating to the head and thorax by 8 d.p.f. This indicates that the mosquitoes efficiently acquired and harbored the virus, enabling its spread beyond the midgut barrier. Immunostaining revealed WNV’s presence in the midgut epithelium at early stages (4 d.p.f.), and by 8 d.p.f., we were able to observe the viruses in the fat body, which underscored the virus’s ability to disseminate to multiple tissues within the mosquito.

Evaluating the transmission potential of WNV from mosquitoes back to vertebrate hosts is a critical aspect of experimental studies on vector competence. Traditional methods, such as the capillary tube technique to capture saliva from infected mosquitoes followed by virus titration in cell culture or RT-qPCR, can underestimate arboviral transmission by mosquitoes [45]. Here, we employed the capillary tube technique to collect saliva from infected mosquitoes, followed by inoculation into naïve mosquitoes. This approach, previously shown to offer greater sensitivity [46], enabled a more precise evaluation of WNV transmission potential. However, to more thoroughly validate the potential of mosquitoes to transmit WNV to a vertebrate host, it will be crucial to conduct further experiments in which AG129 naïve mice are exposed to WNV-infected mosquitoes, followed by testing the mice for infection. Five days post-injection, we analyzed whether these mosquitoes became infected using RT-qPCR. Our approach showed that the saliva of infected mosquitoes exhibited high transmission efficiency, with 95% of samples being infectious to naïve mosquitoes. This confirms the presence of infectious WNV virions in the saliva and underscores the potential for the thesis’s technique to assess transmission rates in *Culex quinquefasciatus* mosquitoes for WNV. While our results demonstrate that AG129 mice are flexible host models for assessing the vector competence of *Culex quinquefasciatus* for WNV in laboratory experiments, there are logistical constraints associated with the use of murine animals. The large number of mice required for these studies and the need to synchronize the age of mosquitoes and mice can be limiting factors. However, the widespread availability of mouse facilities in most research institutions makes the AG129 mouse model more feasible than avian models, such as young chickens, which require additional facilities and extra approvals from animal ethics committees.

## 5. Conclusions

In summary, our findings show that AG129 mice are valuable and versatile models for experimentally evaluating *Culex quinquefasciatus* vector competence for WNV and for exploring various aspects of virus–host interactions. Our study provides comprehensive insights into the transmission dynamics of WNV involving AG129 mice and *Culex quinquefasciatus* mosquitoes. The high susceptibility of AG129 mice to WNV and the efficient transmission of the virus by mosquitoes underscore the suitability of this model for studying WNV transmission and pathogenesis. Replicating the natural complexity of blood feeding on a live vertebrate host enhances the relevance of our findings to real-world transmission scenarios. Overall, the AG129 mouse model, coupled with *Culex quinquefasciatus* mosquitoes, represents a robust system for investigating the vector competence and transmission dynamics of WNV. This model is essential for advancing our understanding of virus–host–vector interactions and for developing effective interventions to mitigate the impact of WNV on public health.

## Data Availability

The data presented in this study are openly available in FigShare dx.doi.org/10.6084/m9.figshare.26096782.

## Supplementary Materials

The following supporting information can be downloaded at https://www.mdpi.com/article/10.3390/tropicalmed9090201/s1, Figure S1: Overview of West Nile Virus: Structure, Transmission Cycles, and Global Distribution.
