# Supplementary material for: Suitable Mouse Model to Study Dynamics of West Nile Virus Infection in Culex quinquefasciatus Mosquitoes
Source: Trop Med Infect Dis. 2024 Sep 2;9(9):201. doi: 10.3390/tropicalmed9090201 (PMC11435581; doi:10.3390/tropicalmed9090201)
Supplement: Supplementary file 1 [file tropicalmed-09-00201-s001.zip › tropicalmed-3101807-supplementary.pdf]

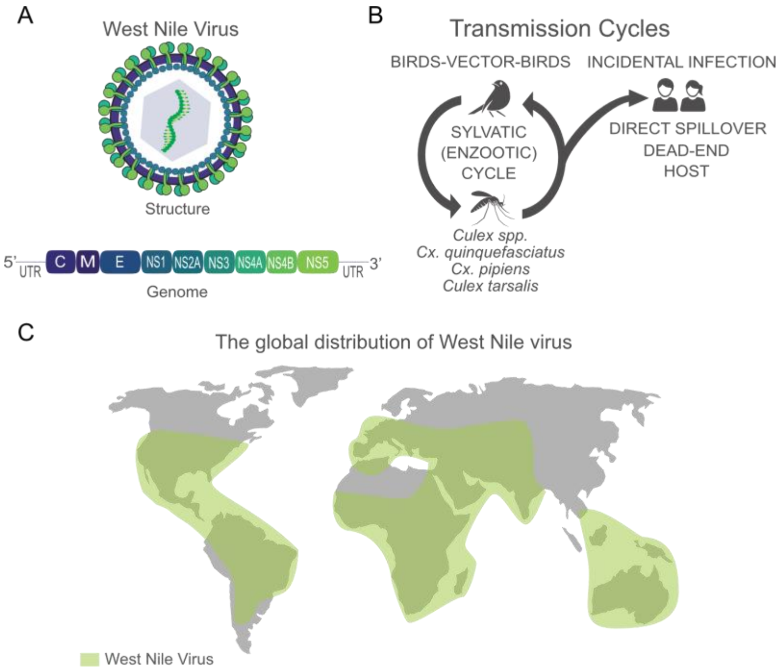

Supplementary Figure S1. Overview of West Nile Virus: Structure (**A**), Transmission Cycles (**B**), and Global Distribution (**C**).
